# Antifungal Drug Susceptibility and Genetic Characterization of Fungi Recovered from COVID-19 Patients

**DOI:** 10.3390/jof7070552

**Published:** 2021-07-11

**Authors:** Milena Kordalewska, Kevin D. Guerrero, Rocio Garcia-Rubio, Cristina Jiménez-Ortigosa, José R. Mediavilla, Marcus H. Cunningham, Frank Hollis, Tao Hong, Kar Fai Chow, Barry N. Kreiswirth, David S. Perlin

**Affiliations:** 1 Center for Discovery and Innovation, Hackensack Meridian Health, Nutley, NJ 07110, USA; kevin.guerrero@hmh-cdi.org (K.D.G.); rocio.garciarubio@hmh-cdi.org (R.G.-R.); cristina.jimenez-ortigosa@hmh-cdi.org (C.J.-O.); jose.mediavilla@hmh-cdi.org (J.R.M.); marcus.cunningham@hmh-cdi.org (M.H.C.); barry.kreiswirth@hmh-cdi.org (B.N.K.); david.perlin@hmh-cdi.org (D.S.P.); 2 Hackensack University Medical Center, Hackensack, NJ 07601, USA; fhollis@optonline.net (F.H.); Tao.Hong@hmhn.org (T.H.); Kar.Chow@hmhn.org (K.F.C.)

**Keywords:** *Candida albicans*, *Candida parapsilosis*, *Aspergillus fumigatus*, antifungal susceptibility, secondary infections, *Candida* pneumonia, candidiasis, aspergillosis, COVID-19-associated candidemia, COVID-19

## Abstract

Fungal infections are common complications of respiratory viral infections and are associated with the increased need for intensive care and elevated mortality. Data regarding microbiological and molecular characteristics of such infections in COVID-19 patients are scarce. Here, we performed a comprehensive analysis, including species identification, antifungal susceptibility testing, molecular resistance determinants analysis, typing, and retrospective clinical data review, of fungal isolates recovered from 19 COVID-19 patients, who were hospitalized at the Hackensack University Medical Center (HUMC) in Hackensack, New Jersey, USA, in the initial phase of the pandemic from April–May 2020. In total, 17 *Candida albicans*, two *C. parapsilosis*, and two *Aspergillus fumigatus* were analyzed. All *Candida* spp. isolates were susceptible to micafungin and azole drugs (fluconazole, voriconazole, posaconazole, itraconazole, isavuconazole). *A. fumigatus* isolates were susceptible to micafungin and all triazole drugs except fluconazole (intrinsic resistance). Multilocus sequence typing (MLST) of *C. albicans* isolates revealed 15 different sequence types (STs), which clustered below the clade-defining limit of p-distance < 0.04. Pulsed-field gel electrophoresis (PFGE) karyotyping revealed no chromosomal rearrangements in these isolates. *A. fumigatus* isolates were of different, non-related genotypes. We speculate that virus- and drug-induced immunosuppression (94.7% of the patients received corticosteroids), together with prolonged hospital stay (median duration of 29 days) and mechanical ventilation (median duration of 24 days) likely increased the susceptibility to secondary respiratory and bloodstream infections in the studied patient population. The presence of fungi in blood or respiratory tract fluid was a prognosticator for poor clinical outcome, which presented as an 89.5% 30-day mortality in our patient cohort.

## 1. Introduction

The emergence and subsequent pandemic of coronavirus disease 2019 (COVID-19), caused by severe acute respiratory syndrome coronavirus 2 (SARS-CoV-2), has led to a global public health crisis with 154.6M+ confirmed cases and 3.2M+ deaths worldwide (as of 6 May 2021) [1]. The relatively high incidence of severe disease and mortality in COVID-19 patients is expected to be enhanced by secondary infections, alongside a lack of natural immunity and viral replication in the lower respiratory tract leading to severe lung injury and acute respiratory distress syndrome (ARDS). Bacterial and fungal infections are common complications of viral pneumonia which lead to an increased need for intensive care and higher mortality rates. Superinfections with *Streptococcus pneumoniae*, *Staphylococcus aureus*, and *Haemophilus influenzae* are well-known complications of severe seasonal and pandemic influenza [2]. More recently, invasive pulmonary aspergillosis (IPA) has been described as a confounder in critically ill patients admitted to the intensive care unit (ICU) with influenza pneumonia [3]. According to the cohort study report by Zheng et al., 20 out of 90 patients with the severe acute respiratory syndrome (SARS) had secondary lower respiratory tract infections in 2003, which accounted for 70.6% of critical SARS patients who underwent an invasive operation. There was a large diversity of pathogens causing secondary infections in SARS patients, with Gram-negative bacteria and *Candida* spp. being the most common ones [4]. Some cases were complicated by invasive fungal disease—aspergillosis [5]—especially in patients receiving corticosteroids [6].

In Wuhan, China, secondary infections have been identified in 14 to 38% of hospitalized patients with COVID-19, of whom, 50% did not survive [7]. In New York City, bacteremia developed in 5.6% of all patients and 11.9% of patients requiring invasive mechanical ventilation [8]. Importantly, researchers also identified a number of secondary fungal co-infections in hospitalized COVID-19 patients [9,10,11,12,13]. A French study found putative IPA in almost one-third of mechanically ventilated patients with COVID-19—a similar prevalence to that observed in patients with influenza [14].

In this context, some authors have speculated that there might exist two non-mutually exclusive explanations for the presence of superinfections in COVID-19 patients [15]. Firstly, severe SARS-CoV-2 infection results in immune system dysregulation [16,17]. Cytokine release syndrome, immune exhaustion, and/or lung damage may leave patients vulnerable to bacterial and/or fungal superinfections [15]. Secondly, critically ill patients, especially those in ICUs and/or receiving mechanical ventilation, are at markedly increased risk for bacterial and fungal infections, independent of COVID-19 [15]. Among COVID-19 ICU patients, mechanical ventilation was reported in 21%–88% of cases [8,18,19,20,21,22].

Given its very recent emergence, data regarding secondary fungal infections in COVID-19 patients are scarce. Thus, it is crucial to understand the clinical and microbiological characteristics of such infections, especially the causal species distribution, the source of infection (nosocomial vs. community-acquired), as well as the antifungal drug susceptibility, to implement appropriate infection prevention measures and/or treatment regimens. Here, we performed a comprehensive analysis (species identification, antifungal susceptibility testing with molecular resistance determinants analysis, and typing) of fungal isolates recovered from COVID-19 patients, who were hospitalized at the Hackensack University Medical Center (HUMC) in Hackensack, NJ, USA.

## 2. Materials and Methods

### 2.1. Fungal Isolates and Culture Conditions

We used a total of 21 clinical isolates of fungi (yeasts, *n* = 19; molds, *n* = 2), which were recovered from specimens (blood, *n* = 11; sputum, *n* = 10) of 19 COVID-19 patients at the Department of Pathology at Hackensack University Medical Center (HUMC) in Hackensack, NJ, USA, in April and May 2020 (Table 1). Yeast and mold isolates were cultured on yeast extract (1%) peptone (2%) dextrose (1%) agar (YPDA), and potato dextrose agar (PDA) plates, respectively, at 37 °C prior to testing. Species identification of all yeast isolates was performed by sequencing the rDNA region (Table 2) and further Nucleotide BLAST (https://blast.ncbi.nlm.nih.gov/Blast.cgi; accessed on 9 June 2020) analysis. Confirmation of *Aspergillus fumigatus* species identification was performed by PCR amplification and sequencing of a portion of the β-tubulin gene (Table 2) followed by Nucleotide BLAST analysis.

### 2.2. DNA Extraction

DNA from fungal isolates was prepared by a 10-min incubation of a single colony in 100 µL of extraction buffer (60 mM sodium bicarbonate [NaHCO_3_], 250 mM potassium chloride [KCl], and 50 mM Tris, pH 9.5) at 95 °C and subsequent addition of 100 µL anti-inhibition buffer (2% bovine serum albumin). After vortex mixing, this DNA-containing solution was used for PCR [23].

### 2.3. Antifungal Susceptibility Testing

Antifungal susceptibility testing for yeast isolates was performed in accordance with the guidelines described in CLSI documents M27 and M60 [24,25]. *C. parapsilosis* ATCC 22019 and *C. krusei* ATCC 6258 were used as quality control strains.

Antifungal susceptibility testing for mold isolates was performed in accordance with the guidelines described in CLSI documents M38 and M61 [26,27]. *A. terreus* ATCC MYA-3633 and *A. flavus* ATCC 204304 were used as quality control strains.

Micafungin (MCF), isavuconazole (ISA) (Astellas Pharma US, Inc., Northbrook, IL, USA), fluconazole (FLC) (LKT Laboratories, Inc., St. Paul, MN, USA), itraconazole (ITR), posaconazole (POS), and voriconazole (VRC) (MilliporeSigma, St. Louis, MO, USA) were obtained as standard powders from their manufacturers, and stock solutions were prepared by dissolving the compounds in 100% dimethyl sulfoxide (DMSO). Minimal inhibitory concentration (MIC) end-points were defined as the lowest drug concentration that caused a prominent decrease (≥50%) in visual growth in relation to the controls. Micafungin minimum effective concentration (MEC) values for molds were read as the lowest drug concentration at which small, rounded, and compact hyphal forms (rosettes) were observed.

### 2.4. Sequencing of FKS1 and ERG11

*FKS1* gene, encoding the echinocandin drug target, and *ERG11* gene, encoding the azole drug target, of all yeast isolates were amplified and sequenced (Table 2). The full coding sequence of the cyp51A gene, including its promoter sequence, was amplified and sequenced (Table 2) using the PCR conditions described before [28]. DNA cyp51A sequences were compared to the cyp51A sequence of the *A. fumigatus* reference strain CBS 144.89 (GenBank accession number AF338659) by using SeqMan Pro 17 (DNASTAR Lasergene).

### 2.5. Karyotyping of C. albicans Isolates

Intact yeast chromosomal DNA was prepared as described by Bai et al. [29]. For karyotyping purposes, the chromosomal DNA bands were separated on 0.8% agarose gels in 1X TAE buffer in a contour-clamped homogeneous electric field (CHEF) electrophoresis system (Bio-Rad, Hercules, CA, USA). Electrophoresis was performed at 2 V/cm for 72 h with initial and final switch times of 2 and 30 min, respectively. The temperature of the running buffer was maintained at 14 °C. After electrophoresis, the gel was stained in ethidium bromide solution (0.5 µg/mL) for 30 min and photographed under UV illumination. *C. albicans* SC5314 and ATCC90028 were used as reference strains, and *Hansenula wingei* (YB-4662-VIA) chromosomal DNA (Bio-Rad, Hercules, CA, USA) was used as a chromosome size marker.

### 2.6. Multilocus Sequence Typing (MLST) of C. albicans Isolates

MLST was performed following a protocol optimized by Bougnoux et al. which comprises an analysis of a set of seven gene fragments: *AAT1a, ACC1, ADP1, MPI, SYA1, VPS13,* and *ZWF1b* [30]. The allelic status (homozygote or heterozygote) of each nucleotide was analyzed using SeqMan Pro 17 (DNASTAR Lasergene). Moreover, the DNA sequences of the seven housekeeping genes got concatenated for each isolate and a dendrogram was constructed based on Clustal Omega alignment using MegAlign Pro 17 (DNASTAR Lasergene). A cutoff P distance of 0.04 was chosen to delimit clusters of closely related strain types [31].

### 2.7. Typing of A. fumigatus Isolates

The *A. fumigatus* isolates included in this study were genotyped following the previously described TRESPERG genotyping assay, which involves sequence analysis of 4 genetic markers: MP-2 antigenic galactomannan protein (MP2; Afu2g05150), a hypothetical protein with a CFEM domain (CFEM; Afu6g14090), cell surface protein A (CSP; Afu3g08990), and putative C-24 sterol reductase (ERG4B; Afu1g07140) (Table 2) [32].

### 2.8. Clinical Data Review

All fungal isolates were de-identified (patient information removed) and given unique HMH BioRepository numbers which enabled to link them to the clinical information. Clinical data, including patient demographics, comorbidities, laboratory tests results, treatment, and outcomes were retrieved from the HMH electronic health record system and reviewed retrospectively. As suggested by Garcia-Vidal et al., infections were defined as community-acquired co-infections if a diagnosis was made at the time of or within the first 24 h of COVID-19 hospital admission. If a diagnosis occurred ≥48 h after admission for COVID-19, these infections were defined as hospital-acquired superinfections [9].

The study received Hackensack University Medical Center Institutional Review Board approval (Study ID: Pro2018-1022 approved on 19 February 2019).

## 3. Results

### 3.1. Species Distribution of Fungal Isolates Recovered from COVID-19 Patients

Twenty-one fungal isolates were cultured from specimens of COVID-19 patients and archived at The Department of Pathology (HUMC) in April and May 2020. Among 19 yeast isolates, 11 isolates recovered from blood were identified to the species level (9/11 *C. albicans*; 2/11 *C. parapsilosis*). Their species were also confirmed by sequencing of rDNA fragments. The remaining 8 isolates, recovered from sputum, were not identified to the species level at the Department of Pathology (HUMC) and labeled as “yeast” or “yeast, not *Cryptococcus*”. These isolates were further identified as *C. albicans* by sequencing of rDNA fragments (Table 1). The two mold isolates recovered from sputum were species-identified as *A. fumigatus*.

### 3.2. Antifungal Susceptibility Testing and Molecular Resistance Determinants Analysis

AFST with echinocandin and azole drugs was performed for all clinical isolates. The MIC results for each isolate are shown in Table 1. All 17 *C. albicans* isolates presented low MIC values (MIC_90_: MCF < 0.03 mg/L; FLC 0.25 mg/L; VRC 0.03 mg/L; ITR 0.03 mg/L; POS < 0.03 mg/L; ISA < 0.03 mg/L). Moreover, sequencing of the gene encoding the echinocandin drug target, *FKS1*, indicated a wild-type genotype of all isolates. Sequencing of the gene encoding the azole drug target, *ERG11*, revealed the presence of several hetero- and homozygous mutations (Table 1). The two *C. parapsilosis* isolates had an MCF MIC value of 2 mg/L and WT *FKS1* genotype, low azole MIC values (same values for both isolates; FLC 0.5 mg/L; VRC 0.125 mg/L; ITR < 0.03 mg/L; POS < 0.03 mg/L; ISA < 0.03 mg/L) and the F215S amino acid change was found in the Erg11. The two *A. fumigatus* isolates were susceptible to MCF (MEC = 0.06 mg/L). These isolates showed high fluconazole MIC value (>64 mg/L), and low MIC values for VRC (0.25 mg/L), ITR (0.5 mg/L), POS (0.03 mg/L), and ISA (0.125 mg/L). Both *A. fumigatus* isolates presented a WT cyp51A genotype, including its promoter.

### 3.3. Relatedness of the Clinical Isolates

**Figure 1:**
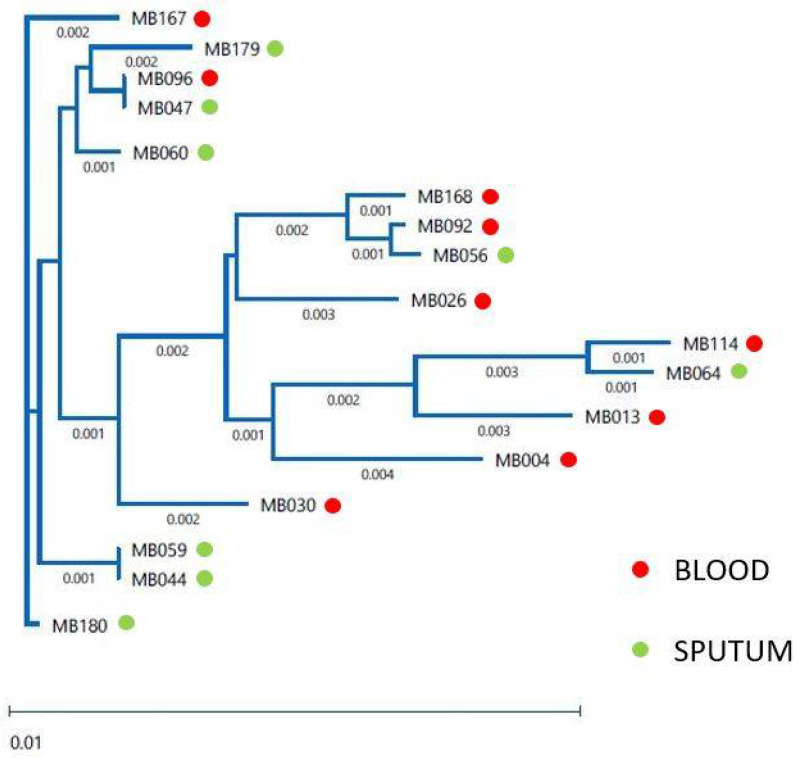
MLST-based dendrogram of 17 *C. albicans* clinical isolates (MB004-MB180) recovered from blood and sputum of COVID-19 patients.

**Figure 2:**
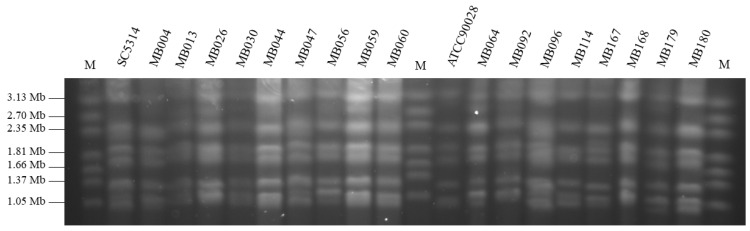
PFGE karyotypes of 17 *Candida albicans* clinical isolates (MB004-MB180) recovered from blood and sputum of COVID-19 patients. M: DNA Size Standard, *Hansenula wingei*; SC5314 & ATCC90028—*Candida albicans* reference strains.

Multilocus sequence typing (MLST), which defines strains as sequence types (STs) based on comparing the sequences at seven house-keeping loci and is the method of choice for molecular typing of many microorganisms, was used for the analysis of the population structure of 17 clinical isolates of *C. albicans*. We determined the presence of 15 different STs. Only two pairs of isolates—MB047 and M096, MB044 and MB059—showed the same STs (Table 3). The relatedness of the *C. albicans* isolates is shown in the dendrogram (Figure 1). All 17 isolates clustered below the clade-defining limit of p-distance < 0.04. Pulsed-field gel electrophoresis (PFGE)-based karyotyping, which separates whole chromosomes, revealed that *C. albicans* genome organization did not vary significantly among the different isolates recovered from the COVID-19 patients as compared to the reference strain SC5314 (Figure 2). TRESPERG typing performed for the *A. fumigatus* isolates showed different genotypes for the four genetic markers tested—isolate MB097 (t04A, m1.1, c13, e07), isolate MB098 (t04A, m3.5, c13, e07).

### 3.4. Clinical Data Review

The results of the clinical data review are presented in Table 4 (Summary of patient characteristics) and Table S1 (Clinical data for each patient). The two pairs of *C. albicans* isolates with the same STs (MB044 and MB059; MB047 and MB096) were recovered from the same patient. The median age of the patients was 60 years, and most of them (68.4%) were of the male sex. The most common comorbidities were hypertension (63.2%) and diabetes (36.8%). All patients (no information for one patient) were ventilated with a median duration of ventilation of 24 days. All patients received antibiotics, 63.2% received antifungals, 94.7% received corticosteroids, and 73.7% received hydroxychloroquine. Antifungal treatment was administered to 6/8 patients with fungal-positive blood culture (MB004; MB030, MB092; MB114; MB167; MB168), 3/3 patients with fungal-positive blood and sputum culture (MB026; MB047 & MB096; MB099), and 3/8 patients with fungal-positive sputum culture (MB056; MB064; MB097). The median day from hospital admission to fungal culture was 14 and 12 for blood and sputum, respectively. The 30-day mortality in this population was 89.5%. The only two of 19 patients that survived had *C. albicans* recovered from sputum and did not receive any antifungal drugs. According to the Garcia-Vidal criteria [9], only one patient’s infection (MB098—*A. fumigatus*) would have been categorized as community-acquired.

## 4. Discussion

A scatter of preliminary studies indicates that secondary bacterial and fungal infections are present in up to 58% of hospitalized patients with severe COVID-19 [7,9,36]. COVID-19-associated pulmonary aspergillosis (CAPA) and COVID-19-associated candidiasis (CAC) were recorded in many countries with reported incidence from 4–35% and 0.7–12.6%, respectively [9,37,38,39,40]. However, in most healthcare settings a somewhat unique diagnostic challenge arose in assessing and managing secondary infections in this population relative to other respiratory viral illnesses. In order to minimize aerosol-generating procedures and healthcare workers’ exposure, most healthcare facilities avoided invasive diagnostic procedures such as bronchoscopy, bronchoalveolar lavage or bronchial wash, and radiologic imaging such as computed tomography (CT) [41]. With such limited diagnostic options, prohibited autopsies, and a lack of definitions for COVID-19-associated secondary diseases in the early days of the pandemic, the true prevalence of secondary fungal infections in the COVID-19 patient population remains unclear.

Here, we based our study on available microbiological material—21 fungal isolates that were recovered from cultures of sputum and blood of 19 severely ill COVID-19 patients at the Department of Pathology at Hackensack University Medical Center (HUMC) in Hackensack, New Jersey, USA. Eleven yeast isolates recovered from blood were identified to the species level as *C. albicans* (9 isolates) and *C. parapsilosis* (2 isolates). Among 10 fungal isolates recovered from sputum, mold isolates were identified as *A. fumigatus*. The remaining 8 yeast isolates were only labeled as “yeast”, or “yeast, not *Cryptococcus*”. It is common that laboratories in the United States do not perform species identification of yeast isolates, especially the non-invasive ones [42]. Ultimately, all these isolates were identified as *C. albicans* by molecular methods. It is noteworthy that *C. auris* was not identified in any of our patients, despite its outbreak status in the State.

*Candida* spp. are isolated from respiratory tract specimens relatively often, especially those obtained from patients on mechanical ventilation. However, the differentiation between colonization and infection is very complicated given no specific diagnostic criteria for ventilator-associated pneumonia exist [43]. It is recommended that *Candida* pneumonia is diagnosed by histopathology [43], which due to the primary disease (COVID-19), was not performed in any of the patients included in the study.

It has been recently speculated that microbiology and antifungal drug resistance patterns will likely be consistent with institutional ecology [15]. Several studies assessed the susceptibility of fungal isolates recovered from COVID-19 patients. Not surprisingly, all *C. auris* isolates were found to be resistant to at least one drug [40,44], whereas no antifungal drug resistance was observed in the majority of isolates from other *Candida* species (*C. albicans, C. glabrata, C. tropicalis*) [40,45,46] and *Saccharomyces cerevisiae* [47]. Single cases of fatal infections caused by drug-resistant *C. glabrata* and *A. fumigatus* were reported in Italy [13] and in the Netherlands [48], respectively.

Hospitals throughout the US have long been struggling to control the use of antibiotics and the many unknowns of COVID-19 have created additional challenges for antimicrobial stewardship programs [49]. Guidelines recommend empirical antibiotics for all patients who are severely ill with suspected COVID-19, and that cessation of therapy is left to the clinicians’ discretion [50]. Not surprisingly, reports to date indicate that antibiotic use is very high (75–100%) among severely ill COVID-19 patients [51,52,53,54]. Moreover, one study from China reported that 7.5% of patients with severe COVID-19 were treated with antifungals [52], while in one hospital in Valencia (Spain), antifungal consumption increased by 15% and 75% in the entire hospital and ICU, respectively [44].

All tested yeast isolates (*C. albicans* and *C. parapsilosis*) presented low echinocandin (MCF) MIC values (categorized as susceptible according to the CLSI breakpoints) and a WT *FKS1* genotype. They also had low azole MIC values (categorized as susceptible according to the CLSI breakpoints for FLC and VRC). Among 10 different *ERG11* genotypes found in *C. albicans*, none of the resulting Erg11 amino acid changes is associated with azole resistance [55,56,57,58]. The amino acid change F215S in Erg11 of *C. parapsilosis* has not been described before but given the low azole MIC values of these isolates, most likely it has no impact on azole susceptibility. The two *A. fumigatus* isolates had MCF MEC of 0.06 mg/L. Given that neither CLSI nor the European Committee on Antimicrobial Susceptibility Testing (EUCAST) provides any recommendation of interpretation for *A. fumigatus* susceptibility testing results with echinocandins (no breakpoints due to the lack of correlation between MEC and outcomes), the available epidemiological cutoff value (ECV ≤ 0.06 mg/L) [59,60] was applied and isolates were categorized as susceptible. *A. fumigatus* isolates had a high fluconazole MIC value (>64 mg/L) since this species is intrinsically resistant to this compound [61], and low MIC values for VRC (0.25 mg/L), ITR (0.5 mg/L), POS (0.03 mg/L), and ISA (0.125 mg/L). All *A. fumigatus* isolates presented a WT *CYP51A* genotype and promoter which agrees with the susceptibility results obtained.

In summary, all 21 fungal isolates were fully susceptible to echinocandin and azole drugs. However, we were not able to assess whether any of the isolates developed antifungal drug resistance during the antifungal treatment (63.2% of patients received antifungals) since no sequential isolates were collected. Moreover, it is unknown if such a susceptibility pattern is characteristic for this health center since no susceptibility data were available for isolates collected in years preceding the pandemic.

Molecular typing can help understand multiple aspects of infectious disease, including pathogen dynamics in a given population, the source/origin of infection, and the relatedness of isolates of the same species [62]. Here, we applied a highly discriminatory and standardized MLST approach for analysis of the population structure of *C. albicans* isolates. We discovered the presence of 15 sequence types (STs), of which 9 were not identified before. Only 3 STs—66, 90, and 485 were previously detected in the US (according to the PubMLST.org database). Identical STs were only obtained when isolates were recovered from different specimens of the same patient. Subsequent concatenation of generated MLST sequences revealed that all 17 *C. albicans* isolates belonged to one cluster (clustered below the clade-defining limit of p-distance < 0.04). Further studies are needed to determine the prevalence of detected STs among *C. albicans* strains infecting HUMC patients and in the general New Jersey population. The karyotypes (the number and size of chromosomes) of the 17 *C. albicans* isolates recovered from the COVID-19 patients revealed no chromosomal rearrangements (copy number variation, loss of heterozygosity, translocations, chromosome truncations) that may occur in response to stresses such as heat shock, host-pathogen interactions including host immunologic responses, and the presence of antifungal drugs [63,64,65]. Since no sequential isolates were collected from the patients, it was not possible to determine if the antifungal therapy provoked changes in the genetic diversity. Collectively, performed typing (MLST and karyotyping) revealed that *C. albicans* isolates recovered from COVID-19 patients were closely related but no clonal spread occurred at HUMC. Analysis of TRESPERG typing results revealed that both *A. fumigatus* isolates were different and non-related based on the genotyping methodology used which was expected as they were isolated from different patients. Since *A. fumigatus* TRESPERG typing is relatively new and has not been broadly implemented in the clinical setting, the determined TRESPERG profiles have never been described before [32,66]. Performed analysis of *Candida* and *Aspergillus* isolates recovered from COVID-19 patients provides new and useful information in the clinical context since it confirms that there was no outbreak of infection or colonization observed in the studied patient population.

Hospitalized patients, critically ill with COVID-19 are at high risk for the development of secondary infections due to the systemic nature, mechanical ventilation, and prolonged hospital and ICU stays associated with severe primary disease. We speculate that these factors played a role in our patient population since the median duration of hospital stay was 29 days (range: 9–81 days) and all patients (no information for one patient) were ventilated with a median duration of ventilation of 24 days (range: 7–81 days). Moreover, COVID-19 patients experience major lung damage (due to viral replication) and immune dysregulation, which is modulated by the use of corticosteroids, anti–IL-6 monoclonal antibodies (tocilizumab, sarilumab), or other immunomodulatory agents [15]. Here, 94.7% of the patients received corticosteroids. Additionally, one patient received sarilumab. A combination of virus- and drug-induced immunosuppression likely increased the susceptibility to secondary infections. The 30-day mortality in this population was 89.5%, which is higher than the overall reported mortality of 68.5% (217/317) from ventilated patients at HUMC in the comparable time period [67]. The elevated mortality rate in COVID-19 patients with fungal superinfections, irrespective of the drug resistance profile of the infecting organism, is in agreement with other reports on COVID-19-associated candidemia [11,12,68].

We recognize several limitations of our study, in which we analyzed only a small number of fungal isolates (*n* = 21) due to the research restrictions implemented at HUMC in the initial phase of the COVID-19 pandemic (April–May 2020). Given the diagnostic restrictions, we cannot exclude that one of the recovered *Aspergillus* isolates (MB098) represented environmental contamination rather than a causative agent of infection. Similarly, despite the assumption that all the sputum samples represent lower respiratory tract flora rather than flora in the mouth (all patients were ventilated, and sputum samples were collected through suction) but lacking histopathology data, we do not have a definite clinical implication for *C. albicans* presence in the sputum samples. However, the presence of fungi in blood or respiratory tract fluid was an indicator of the patient’s severe condition and a prognosticator for poor clinical outcome, which presented as an 89.5% 30-day mortality in our patient cohort. Such observation is consistent with the results of a study performed in Brazil, where mechanically-ventilated patients with positive cultures were 3.8 times more likely to die than those without superinfections [69]. Lastly, we were not able to assess whether any of the isolates developed antifungal drug resistance during the antifungal treatment and whether the susceptibility pattern is characteristic for this health center. Given the existing data on the high mortality of COVID-19-associated secondary infections, preparing for the prevention, diagnosis, and treatment of these infections is an important adjunct to addressing COVID-19 and related respiratory viral diseases such as influenza.

## Figures and Tables

**Table 1:** Results of identification, antifungal susceptibility testing, and antifungal drug target sequencing for fungal isolates recovered from COVID-19 patients.

Isolate	Specimen	Identification		MIC [mg/L] *						*FKS1*	Erg11/Cyp51A
		Original (at HUMC)	Species Confirmation (rDNA seq)	MCF	FLC	VRC	ITR	POS	ISA		
MB 004	Blood	*C. albicans*	*C. albicans*	<0.03	<0.25	0.03	<0.03	<0.03	<0.03	WT	E266D
MB 013	Blood	*C. albicans*	*C. albicans*	<0.03	0.25	0.03	0.03	0.03	0.03	WT	V437I
MB 026	Blood	*C. albicans*	*C. albicans*	<0.03	0.25	0.03	<0.03	<0.03	<0.03	WT	E266D, H283H/L, K342K/R
MB 030	Blood	*C. albicans*	*C. albicans*	<0.03	0.25	0.03	0.03	<0.03	<0.03	WT	V437V/I
MB 044	Sputum	yeast	*C. albicans*	<0.03	0.25	0.03	0.03	<0.03	<0.03	WT	K128K/T
MB 047	Sputum	yeast	*C. albicans*	<0.03	0.25	0.03	0.03	<0.03	<0.03	WT	K128K/T
MB 056	Sputum	yeast	*C. albicans*	<0.03	0.25	0.03	0.03	<0.03	<0.03	WT	V437V/I
MB 059	Sputum	yeast (not *Cryptococcus*)	*C. albicans*	<0.03	0.25	0.03	0.03	<0.03	<0.03	WT	K128K/T
MB 060	Sputum	yeast	*C. albicans*	<0.03	0.25	0.03	0.03	<0.03	<0.03	WT	K128K/T
MB 064	Sputum	yeast (not *Cryptococcus*)	*C. albicans*	<0.03	0.25	0.03	0.03	<0.03	<0.03	WT	D116D/E, E266E/D
MB 092	Blood	*C. albicans*	*C. albicans*	<0.03	<0.25	0.03	0.03	<0.03	<0.03	WT	V437V/I
MB 096	Blood	*C. albicans*	*C. albicans*	<0.03	0.25	0.03	0.03	<0.03	<0.03	WT	D116E, K128K/T
MB 114	Blood	*C. albicans*	*C. albicans*	<0.03	0.25	0.03	0.03	<0.03	<0.03	WT	E266E/D, V437V/I
MB 167	Blood	*C. albicans*	*C. albicans*	<0.03	0.25	0.03	0.03	<0.03	<0.03	WT	D116E, K128T
MB 168	Blood	*C. albicans*	*C. albicans*	<0.03	0.25	0.03	0.03	<0.03	<0.03	WT	V437V/I
MB 179	Sputum	yeast (not *Cryptococcus*)	*C. albicans*	<0.03	0.25	0.03	0.03	<0.03	<0.03	WT	D116D/E, K128K/T, V159V/I
MB 180	Sputum	yeast (not *Cryptococcus*)	*C. albicans*	<0.03	<0.25	0.06	<0.03	<0.03	<0.03	WT	D116D/E, K128K/T
MB 025	Blood	*C. parapsilosis*	*C. parapsilosis*	2	0.5	0.125	<0.03	<0.03	<0.03	WT **	F215S
MB 099	Blood	*C. parapsilosis*	*C. parapsilosis*	2	0.5	0.125	<0.03	<0.03	<0.03	WT **	F215S
MB 097	Sputum	*A. fumigatus*	*A. fumigatus*	0.06	>64	0.25	0.5	0.03	0.125	N/A	WT
MB 098	Sputum	*A. fumigatus*	*A. fumigatus*	0.06	>64	0.25	0.5	0.03	0.125	N/A	WT

* MEC in case of MCF and *A. fumigatus* clinical isolates. ** *C. parapsilosis* WT contains a naturally occurring polymorphism in the last amino acid of the hot spot 1.

**Table 2:** Primers used in the study.

Gene	Organism	Primer Name	Sequence	Use	Reference
*rDNA*	*Candida* spp.	Fun-rDNAF	GGTCATTTAGAGGAAGTAAAAGTCG	PCR + seq	S. Katiyar, personal communication
		Fun-rDNAR	YGATATGCTTAAGTTCAGCGGGTA	PCR + seq	
*β-tubulin*	* Aspergillus * spp.	btub2F	TTCACCTTCAGACCGGT	PCR + seq	[33]
		btub4R	AGTTGTCGGGACGGAATAG	PCR + seq	
*FKS1*	*C. albicans*	F2426	CATTGCTGTGGCCACTTTAG	PCR + seq	[34]
		R2919	GATTTCCATTTCCGTGGTAGC	PCR	
		F4590	TACTATGGTCATCCAGGTTTCC	PCR + seq	
		R4954	GGTCAAATCAGTGAAAACCG	PCR	
	*C. parapsilosis*	CparF	CTCCAAGTCCTCATATGCAC	PCR + seq	
		CparR	AGATGTTTCTCCATGGTGTC	PCR	
		F4500	AAGATTGGTGCTGGTATGGG	PCR + seq	[35]
		R5112	TAATGGTGCTTGCCAATGAG	PCR	
*ERG11*	*C. albicans*	Ca_ERG11_F	ATG GCT ATT GTT GAA ACT GTC ATT G	PCR	This study *
		Ca_ERG11_R	TTA AAA CAT ACA AGT TTC TCT TTT TTC CC	PCR	
		Ca_ERG11_731-750_F	GGA GAC GTG ATG CTG CTC AA	seq	
		Ca_ERG11_918-938_R	GCA GAA GTA TGT TGA CCA CCC	seq	
	*C. parapsilosis*	Cpara_erg11_F1	TCCCTACCTTCGTTCATC	PCR + seq	
		Cpara_erg11_R1	CGAGGTGAGTCAACAAAG	PCR + seq	
		Cpara_erg11_F2	AGAGACGGGTGACATTG	seq	
		Cpara_erg11_R2	TGGCACTAGTATGCTGTC	seq	
*CYP51A* promoter	*A. fumigatus*	A5	CTT TTT CGA CTG CCG CGC	PCR + seq	[28]
		A7	TCA TAT GTT GCT CAG CGG	PCR + seq	
*CYP51A*	*A. fumigatus*	P450.1	ATG GTG CCG ATG CTA TGG	PCR	
		P450.2	CTG TCT CAC TTG GAT GTG	PCR	
		CypA1	CTT ACG GCC TAC ATG GCC	seq	
		CypA2	TTC GAC CGC TTC TCC CAG	seq	
		A3	TAG TCC ATT GAC GAC CCC	seq	
TRESPERG genotyping	*A. fumigatus*	CSP1F	TTGGGTGGCATTGTGCCAA	PCR + seq	[32]
		CSP2R	GAGCATGACAACCCAGATACCA	PCR + seq	
		MP2A	ATGCGGTTCTCTGCGTTA	PCR	
		MP2B	CAGCAACAGTGCAAATGC	PCR	
		MP2_P1	CTCGAACTTGGCTACGAC	seq	
		MP2_P2	AGGTAGTGGAGGTCACTG	seq	
		CFEMA	ATGAAGGCCTCTGTGTC	PCR + seq	
		CFEMB	AGGATAATCAAGGCAGCG	PCR + seq	
		ERG4B_P1	ATGACTGTCACACGCTCC	PCR + seq	
		ERG4B_P2	TAGACGGCACCAATCCAC	PCR + seq	

* Primers were designed on the basis of reference *ERG11* sequences: *C. albicans*—C5_00660C_A; *C. parapsilosis*—CPAR2_303740.

**Table 3:** Results of multilocus sequence typing (MLST) of *Candida albicans* isolates.

Isolate	Specimen	*AAT1a*	*ACC1*	*ADP1*	*MPI*	*SYA1*	*VPS13*	*ZWF1b*	ST
MB004	Blood	43	14	8	4	7	10	8	927
MB013	Blood	117	7	21	34	* 238	24	6	New ST, A
MB026	Blood	25	7	6	3	6	27	37	90
MB030	Blood	20	3	6	2	51	132	5	New ST, B
MB044	Sputum	2	5	5	2	2	6	20	485
MB047	Sputum	8	5	* 6	2	2	6	5	New ST, C
MB056	Sputum	33	14	38	2	* 136	122	15	New ST, D
MB059	Sputum	2	5	5	2	2	6	20	485
MB060	Sputum	8	3	6	2	2	6	49	New ST, E
MB064	Sputum	13	7	15	6	7	55	15	1830
MB092	Blood	33	7	6	2	78	122	15	New ST, F
MB096	Blood	8	5	* 6	2	2	6	5	New ST, C
MB114	Blood	13	10	15	6	7	15	15	New ST, G
MB167	Blood	2	2	5	2	2	68	5	New ST, H
MB168	Blood	33	3	38	2	78	122	22	New ST, I
MB179	Sputum	8	2	5	9	2	6	5	285
MB180	Sputum	2	3	5	2	2	6	5	66

ST, sequence type. * Closest match.

**Table 4:** The main characteristics of patients whose fungal isolates were recovered from data and are shown as median (IQR) [range] or *n* (%).

Characteristic	Values
Age	60 (55–69) (49–84)
**BMI**	29.3 (24.96–37.34) (22.91–58.5)
≥30	8 (42.1%)
**Sex**	
Male	13 (68.4%)
Female	6 (31.6%)
**Comorbidities**	
Diabetes	7 (36.8%)
Asthma	3 (15.8%)
COPD	1 (5.3%)
Hypertension	12 (63.2%)
Cancer	0
Hypercholesterolemia	5 (26.3%)
Hyperlipidemia	2 (10.5%)
Arthritis	4 (21.1%)
**Treatment**	
Antibiotics	19 (100%)
Antifungals	12 (63.2%)
Corticosteroids	18 (94.7%)
Hydroxychloroquine	14 (73.7%)
Remdesivir	3 (15.8%)
Lopinavir-ritonavir	1 (5.3%)
Sarilumab	1 (5.3%)
**Fungal culture**	
Day of the first blood culture	14 (11–19) (8–28)
Day of the first sputum culture	12 (6–18) (1–40)
Fungal culture only	6 (31.6%)
Fungal and bacterial culture	13 (68.4%)
**Hospitalization**	
Days of hospital stay	29 (15–33) (9–81)
ICU admission	6 (31.6%)
Days in ICU	24 (8.25–41.25) (0–54)
Ventilation	18 (94.7%) *
Days on ventilator	24 (11–31.25) (7–81)
**Death**	17 (89.5%)
Respiratory failure	14/17 (82.3%)
Cardiac failure	2/17 (11.8%)
No data	1/17 (5.9%)

* no data available for one patient.

## Data Availability

All data are available within the article and Supplementary Materials.

## Supplementary Materials

The following are available online at https://www.mdpi.com/article/10.3390/jof7070552/s1, Table S1: Clinical data for each patient.

## References

[1] WHO WHO Coronavirus Disease (COVID-19) Dashboard. https://covid19.who.int/.

[2] Liderot K., Ahl M., Ozenci V. (2013). Secondary bacterial infections in patients with seasonal influenza A and pandemic H1N1. BioMed Res. Int..

[3] Rijnders B.J.A., Schauwvlieghe A., Wauters J. (2020). Influenza-associated pulmonary aspergillosis: A local or global lethal combination?. Clin. Infect. Dis..

[4] Zheng Z.G., Chen R.C., Li Y.M. (2003). The clinical characteristics of secondary infection of lower respiratory in severe acute respiratory syndrome. Chin. J. Respir. Crit. Care Med..

[5] Hwang D.M., Chamberlain D.W., Poutanen S.M., Low D.E., Asa S.L., Butany J. (2005). Pulmonary pathology of severe acute respiratory syndrome in Toronto. Mod. Pathol..

[6] Wu Y.P., Wei R., Verhoef J. (2003). Real time assay of Aspergillus should be used in SARS patients receiving corticosteroids. BMJ.

[7] Zhou F., Yu T., Du R., Fan G., Liu Y., Liu Z., Xiang J., Wang Y., Song B., Gu X. (2020). Clinical course and risk factors for mortality of adult inpatients with COVID-19 in Wuhan, China: A retrospective cohort study. Lancet.

[8] Goyal P., Choi J.J., Pinheiro L.C., Schenck E.J., Chen R., Jabri A., Satlin M.J., Campion T.R., Nahid M., Ringel J.B. (2020). Clinical Characteristics of Covid-19 in New York City. N. Engl. J. Med..

[9] Garcia-Vidal C., Sanjuan G., Moreno-Garcia E., Puerta-Alcalde P., Garcia-Pouton N., Chumbita M., Fernandez-Pittol M., Pitart C., Inciarte A., Bodro M. (2020). Incidence of co-infections and superinfections in hospitalized patients with COVID-19: A retrospective cohort study. Clin. Microbiol. Infect..

[10] Nucci M., Barreiros G., Guimaraes L.F., Deriquehem V.A.S., Castineiras A.C., Nouer S.A. (2021). Increased incidence of candidemia in a tertiary care hospital with the COVID-19 pandemic. Mycoses.

[11] Mastrangelo A., Germinario B.N., Ferrante M., Frangi C., Li Voti R., Muccini C., Ripa M., COVID-BioB Study Group (2020). Candidemia in COVID-19 patients: Incidence and characteristics in a prospective cohort compared to historical non-COVID-19 controls. Clin. Infect. Dis..

[12] Villanueva-Lozano H., Trevino-Rangel R.J., Gonzalez G.M., Ramirez-Elizondo M.T., Lara-Medrano R., Aleman-Bocanegra M.C., Guajardo-Lara C.E., Gaona-Chavez N., Castilleja-Leal F., Torre-Amione G. (2021). Outbreak of Candida auris infection in a COVID-19 hospital in Mexico. Clin. Microbiol. Infect..

[13] Posteraro B., Torelli R., Vella A., Leone P.M., De Angelis G., De Carolis E., Ventura G., Sanguinetti M., Fantoni M. (2020). Pan-Echinocandin-Resistant Candida glabrata Bloodstream Infection Complicating COVID-19: A Fatal Case Report. J. Fungi.

[14] Alanio A., Delliere S., Fodil S., Bretagne S., Megarbane B. (2020). Prevalence of putative invasive pulmonary aspergillosis in critically ill patients with COVID-19. Lancet Respir. Med..

[15] Clancy C.J., Nguyen M.H. (2020). COVID-19, superinfections and antimicrobial development: What can we expect?. Clin. Infect. Dis..

[16] Chen G., Wu D., Guo W., Cao Y., Huang D., Wang H., Wang T., Zhang X., Chen H., Yu H. (2020). Clinical and immunological features of severe and moderate coronavirus disease 2019. J. Clin. Investig..

[17] Huang C., Wang Y., Li X., Ren L., Zhao J., Hu Y., Zhang L., Fan G., Xu J., Gu X. (2020). Clinical features of patients infected with 2019 novel coronavirus in Wuhan, China. Lancet.

[18] Yang X., Yu Y., Xu J., Shu H., Xia J., Liu H., Wu Y., Zhang L., Yu Z., Fang M. (2020). Clinical course and outcomes of critically ill patients with SARS-CoV-2 pneumonia in Wuhan, China: A single-centered, retrospective, observational study. Lancet Respir. Med..

[19] Du Y., Tu L., Zhu P., Mu M., Wang R., Yang P., Wang X., Hu C., Ping R., Hu P. (2020). Clinical Features of 85 Fatal Cases of COVID-19 from Wuhan. A Retrospective Observational Study. Am. J. Respir. Crit. Care Med..

[20] Arentz M., Yim E., Klaff L., Lokhandwala S., Riedo F.X., Chong M., Lee M. (2020). Characteristics and Outcomes of 21 Critically Ill Patients With COVID-19 in Washington State. JAMA.

[21] Wang D., Hu B., Hu C., Zhu F., Liu X., Zhang J., Wang B., Xiang H., Cheng Z., Xiong Y. (2020). Clinical Characteristics of 138 Hospitalized Patients With 2019 Novel Coronavirus-Infected Pneumonia in Wuhan, China. JAMA.

[22] Grasselli G., Zangrillo A., Zanella A., Antonelli M., Cabrini L., Castelli A., Cereda D., Coluccello A., Foti G., Fumagalli R. (2020). Baseline Characteristics and Outcomes of 1591 Patients Infected With SARS-CoV-2 Admitted to ICUs of the Lombardy Region, Italy. JAMA.

[23] Brillowska-Dabrowska A., Nielsen S.S., Nielsen H.V., Arendrup M.C. (2010). Optimized 5-hour multiplex PCR test for the detection of tinea unguium: Performance in a routine PCR laboratory. Med. Mycol..

[24] CLSI (2017). Reference Method for Broth Dilution Antifungal Susceptibility Testing of Yeast. CLSI Standard M27.

[25] CLSI (2020). Performance Standards for Antifungal Susceptibility Testing of Yeasts. CLSI Supplement M60.

[26] CLSI (2017). Reference Method for Broth Dilution Antifungal Susceptibility Testing of Filamentous Fungi. CLSI Standard M38.

[27] CLSI (2020). Performance Standards for Antifungal Susceptibility Testing of Filamentous Fungi. CLSI Supplement M61.

[28] Mellado E., Garcia-Effron G., Alcazar-Fuoli L., Melchers W.J., Verweij P.E., Cuenca-Estrella M., Rodriguez-Tudela J.L. (2007). A new Aspergillus fumigatus resistance mechanism conferring in vitro cross-resistance to azole antifungals involves a combination of cyp51A alterations. Antimicrob. Agents Chemother..

[29] Bai F.Y., Liang H.Y., Jia J.H. (2000). Taxonomic relationships among the taxa in the Candida guilliermondii complex, as revealed by comparative electrophoretic karyotyping. Int. J. Syst. Evol. Microbiol..

[30] Bougnoux M.E., Tavanti A., Bouchier C., Gow N.A., Magnier A., Davidson A.D., Maiden M.C., D’Enfert C., Odds F.C. (2003). Collaborative consensus for optimized multilocus sequence typing of Candida albicans. J. Clin. Microbiol..

[31] Odds F.C., Bougnoux M.E., Shaw D.J., Bain J.M., Davidson A.D., Diogo D., Jacobsen M.D., Lecomte M., Li S.Y., Tavanti A. (2007). Molecular phylogenetics of Candida albicans. Eukaryot. Cell.

[32] Garcia-Rubio R., Escribano P., Gomez A., Guinea J., Mellado E. (2018). Comparison of Two Highly Discriminatory Typing Methods to Analyze Aspergillus fumigatus Azole Resistance. Front. Microbiol..

[33] Alcazar-Fuoli L., Mellado E., Alastruey-Izquierdo A., Cuenca-Estrella M., Rodriguez-Tudela J.L. (2008). Aspergillus section Fumigati: Antifungal susceptibility patterns and sequence-based identification. Antimicrob. Agents Chemother..

[34] Arendrup M.C., Garcia-Effron G., Lass-Florl C., Lopez A.G., Rodriguez-Tudela J.L., Cuenca-Estrella M., Perlin D.S. (2010). Echinocandin susceptibility testing of Candida species: Comparison of EUCAST EDef 7.1, CLSI M27-A3, Etest, disk diffusion, and agar dilution methods with RPMI and isosensitest media. Antimicrob. Agents Chemother..

[35] Garcia-Effron G., Katiyar S.K., Park S., Edlind T.D., Perlin D.S. (2008). A naturally occurring proline-to-alanine amino acid change in Fks1p in Candida parapsilosis, Candida orthopsilosis, and Candida metapsilosis accounts for reduced echinocandin susceptibility. Antimicrob. Agents. Chemother..

[36] Zhang H., Zhang Y., Wu J., Li Y., Zhou X., Li X., Chen H., Guo M., Chen S., Sun F. (2020). Risks and features of secondary infections in severe and critical ill COVID-19 patients. Emerg. Microbes Infect..

[37] Lamoth F., Glampedakis E., Boillat-Blanco N., Oddo M., Pagani J.L. (2020). Incidence of invasive pulmonary aspergillosis among critically ill COVID-19 patients. Clin. Microbiol. Infect..

[38] Rutsaert L., Steinfort N., Van Hunsel T., Bomans P., Naesens R., Mertes H., Dits H., Van Regenmortel N. (2020). COVID-19-associated invasive pulmonary aspergillosis. Ann. Intensive Care.

[39] White P.L., Dhillon R., Cordey A., Hughes H., Faggian F., Soni S., Pandey M., Whitaker H., May A., Morgan M. (2020). A national strategy to diagnose COVID-19 associated invasive fungal disease in the ICU. Clin. Infect. Dis..

[40] Chowdhary A., Tarai B., Singh A., Sharma A. (2020). Multidrug-Resistant Candida auris Infections in Critically Ill Coronavirus Disease Patients, India, April-July 2020. Emerg. Infect. Dis..

[41] Peman J., Ruiz-Gaitan A., Garcia-Vidal C., Salavert M., Ramirez P., Puchades F., Garcia-Hita M., Alastruey-Izquierdo A., Quindos G. (2020). Fungal co-infection in COVID-19 patients: Should we be concerned?. Rev. Iberoam. Micol..

[42] Lockhart S.R., Jackson B.R., Vallabhaneni S., Ostrosky-Zeichner L., Pappas P.G., Chiller T. (2017). Thinking beyond the Common Candida Species: Need for Species-Level Identification of Candida Due to the Emergence of Multidrug-Resistant Candida auris. J. Clin. Microbiol..

[43] Liu J., Yu Y.T., Xu C.H., Chen D.C. (2020). Candida Colonization in the Respiratory Tract: What Is the Significance?. Front. Med..

[44] Mulet Bayona J.V., Tormo Palop N., Salvador Garcia C., Fuster Escriva B., Chanza Avino M., Ortega Garcia P., Gimeno Cardona C. (2021). Impact of the SARS-CoV-2 Pandemic in Candidaemia, Invasive Aspergillosis and Antifungal Consumption in a Tertiary Hospital. J. Fungi.

[45] Al-Hatmi A.M.S., Mohsin J., Al-Huraizi A., Khamis F. (2021). COVID-19 associated invasive candidiasis. J. Infect..

[46] Gorkem A., Sav H., Kaan O., Eren E. (2021). Coronavirus disease and candidemia infection: A case report. J. Mycol. Med..

[47] Ventoulis I., Sarmourli T., Amoiridou P., Mantzana P., Exindari M., Gioula G., Vyzantiadis T.A. (2020). Bloodstream Infection by Saccharomyces cerevisiae in Two COVID-19 Patients after Receiving Supplementation of Saccharomyces in the ICU. J. Fungi.

[48] Meijer E.F.J., Dofferhoff A.S.M., Hoiting O., Buil J.B., Meis J.F. (2020). Azole-Resistant COVID-19-Associated Pulmonary Aspergillosis in an Immunocompetent Host: A Case Report. J. Fungi.

[49] Martin E., Philbin M., Hughes G., Bergin C., Talento A.F. (2021). Antimicrobial stewardship challenges and innovative initiatives in the acute hospital setting during the COVID-19 pandemic. J. Antimicrob. Chemother..

[50] Adler H., Ball R., Fisher M., Mortimer K., Vardhan M.S. (2020). Low rate of bacterial co-infection in patients with COVID-19. Lancet Microbe.

[51] Chen T., Wu D., Chen H., Yan W., Yang D., Chen G., Ma K., Xu D., Yu H., Wang H. (2020). Clinical characteristics of 113 deceased patients with coronavirus disease 2019: Retrospective study. BMJ.

[52] Guan W.J., Ni Z.Y., Hu Y., Liang W.H., Ou C.Q., He J.X., Liu L., Shan H., Lei C.L., Hui D.S.C. (2020). Clinical Characteristics of Coronavirus Disease 2019 in China. N. Engl. J. Med..

[53] Cox M.J., Loman N., Bogaert D., O’Grady J. (2020). Co-infections: Potentially lethal and unexplored in COVID-19. Lancet Microbe.

[54] Zhou P., Liu Z., Chen Y., Xiao Y., Huang X., Fan X.G. (2020). Bacterial and fungal infections in COVID-19 patients: A matter of concern. Infect. Control Hosp. Epidemiol..

[55] Perea S., Lopez-Ribot J.L., Kirkpatrick W.R., McAtee R.K., Santillan R.A., Martinez M., Calabrese D., Sanglard D., Patterson T.F. (2001). Prevalence of molecular mechanisms of resistance to azole antifungal agents in Candida albicans strains displaying high-level fluconazole resistance isolated from human immunodeficiency virus-infected patients. Antimicrob. Agents Chemother..

[56] Flowers S.A., Colon B., Whaley S.G., Schuler M.A., Rogers P.D. (2015). Contribution of clinically derived mutations in ERG11 to azole resistance in Candida albicans. Antimicrob. Agents Chemother..

[57] Ying Y., Zhao Y., Hu X., Cai Z., Liu X., Jin G., Zhang J., Zhang J., Liu J., Huang X. (2013). In vitro fluconazole susceptibility of 1,903 clinical isolates of Candida albicans and the identification of ERG11 mutations. Microb. Drug Resist..

[58] Xu Y., Chen L., Li C. (2008). Susceptibility of clinical isolates of Candida species to fluconazole and detection of Candida albicans ERG11 mutations. J. Antimicrob. Chemother..

[59] Pfaller M.A., Boyken L., Hollis R.J., Kroeger J., Messer S.A., Tendolkar S., Diekema D.J. (2009). In vitro susceptibility of clinical isolates of Aspergillus spp. to anidulafungin, caspofungin, and micafungin: A head-to-head comparison using the CLSI M38-A2 broth microdilution method. J. Clin. Microbiol..

[60] Pfaller M.A., Boyken L., Hollis R.J., Kroeger J., Messer S.A., Tendolkar S., Diekema D.J. (2010). Wild-type minimum effective concentration distributions and epidemiologic cutoff values for caspofungin and Aspergillus spp. as determined by Clinical and Laboratory Standards Institute broth microdilution methods. Diagn. Microbiol. Infect. Dis..

[61] Leonardelli F., Macedo D., Dudiuk C., Cabeza M.S., Gamarra S., Garcia-Effron G. (2016). Aspergillus fumigatus Intrinsic Fluconazole Resistance Is Due to the Naturally Occurring T301I Substitution in Cyp51Ap. Antimicrob. Agents Chemother..

[62] McManus B.A., Coleman D.C. (2014). Molecular epidemiology, phylogeny and evolution of Candida albicans. Infect. Genet. Evol..

[63] Selmecki A., Forche A., Berman J. (2006). Aneuploidy and isochromosome formation in drug-resistant Candida albicans. Science.

[64] Bouchonville K., Forche A., Tang K.E., Selmecki A., Berman J. (2009). Aneuploid chromosomes are highly unstable during DNA transformation of Candida albicans. Eukaryot. Cell.

[65] Forche A., Magee P.T., Selmecki A., Berman J., May G. (2009). Evolution in Candida albicans populations during a single passage through a mouse host. Genetics.

[66] Fan H., Chen Y., Duan L., Zhao J., Qin C., Li H., Sun J., Han L. (2020). Comparison of Two Typing Methods for Characterization of Azole Resistance in Aspergillus fumigatus from Potting Soil Samples in a Chinese Hospital. Antimicrob. Agents Chemother..

[67] Donato M.L., Park S., Baker M., Korngold R., Morawski A., Geng X., Tan M., Ip A., Goldberg S., Rowley S. (2021). Clinical and laboratory evaluation of patients with SARS-CoV-2 pneumonia treated with high-titer convalescent plasma. JCI Insight.

[68] Arastehfar A., Shaban T., Zarrinfar H., Roudbary M., Ghazanfari M., Hedayati M.T., Sedaghat A., Ilkit M., Najafzadeh M.J., Perlin D.S. (2021). Candidemia among Iranian Patients with Severe COVID-19 Admitted to ICUs. J. Fungi.

[69] Silva D.L., Lima C.M., Magalhaes V.C.R., Baltazar L.M., Peres N.T.A., Caligiorne R.B., Moura A.S., Fereguetti T., Martins J.C., Rabelo L.F. (2021). Fungal and bacterial coinfections increase mortality of severely ill COVID-19 patients. J. Hosp. Infect..

